# Low coverage of influenza vaccination among Chinese children aged 12-23 months: Prevalence and associated factors

**DOI:** 10.1371/journal.pone.0205561

**Published:** 2018-10-10

**Authors:** Joseph T. F. Lau, Catalina S. M. Ng, Anise M. S. Wu, Yee Ling Ma, Mason M. C. Lau

**Affiliations:** 1 Centre for Health Behaviours Research, The Jockey Club School of Public Health and Primary Care, Faculty of Medicine, The Chinese University of Hong Kong, New Territories, Hong Kong, China; 2 Department of Early Childhood Education, Education University of Hong Kong, Taipo, New Territories, Hong Kong, China; 3 Department of Psychology, Faculty of Social Sciences, University of Macau, Macao, China; Public Health England, UNITED KINGDOM

## Abstract

This study aimed to investigate prevalence and associated factors of influenza vaccination (IV) among children aged 12–23 months. Our cross-sectional survey interviewed 489 parents of children aged 12–23 months anonymously at twelve maternal and child health centers in Hong Kong. Results showed that only 11.5% of the children had ever received IV (64.3% being subsidized). Adjusted for the child’s age, significant factors of the children’s IV included parental knowledge about governmental policy/recommendation (Adjusted odds ratio [AOR] = 2.64, 95%CI = 1.09,6.40), knowledge about annual IV requirement (AOR = 2.30, 95%CI = 1.21,4.38), perceived safety-related barrier (AOR≥0.14, 95%CI = 0.06,0.33), cue to action (AOR = 7.79, 95%CI = 3.45,17.58), and subjective norm (AOR = 4.59, 95%CI = 2.34,9.00). IV prevalence of children aged 12–23 months remained low despite a subsidization scheme. The higher IV prevalence of older children reported by other studies suggested that parents postponed action. Promotion campaigns should shift emphases from cost reduction and mass media approaches to dissemination of knowledge about IV policy and safety, enhancement of health professionals’ advice, and creation of supportive subjective norm.

## Introduction

Influenza causes high risk of complications such as pneumonia and bronchitis, which may lead to hospitalizations or even deaths among young children [1,2]. The World Health Organization recommends annual influenza vaccination (IV) to children aged 6–59 months [3]; the Advisory Committee for Immunization Practices in the U.S. also recommended it to children aged 6–23 (2004) and 24–59 months (2006) [4]. Age is important as prevalence of IV concerning <2 or ≥2 years old children differ. Previous studies in the U.S. reported IV prevalence specifically for <2 years old children (56% in 2003 and 31.9% in 2006) [5,6]. Other studies conducted in the U.S. reported IV prevalence both for children aged 6–23 (41.0%-74.4%) and 24–59 months (range = 36%-53.3%) [7–9]. In Spain, there was no noticeable difference between the two age groups (2.2% versus 5.2%) [10]; the younger group showed lower prevalence than the older group of children in Poland (4.1% in 0–1 year, 26.4% in 1–2 years, and 42.3% in 2–5 years) [11]; reverse trends were observed in the U.S. (56% versus 38%) [12] and France (21% versus 15%) [13].

Both sociocultural factors such as policies (e.g., IV providers’ visits to schools) and experience related to IV are important in explaining age differences [8,9]. The factors of IV may differ between <2 and >2 years old children, although no study has directly compared such between-group differences. Different strategies might hence be required to promote IV for the two age groups. While there are a number of studies conducted among children >2 years old and reported factors such as perceived vaccine effectiveness, perceived influenza severity, and subjective norm [14–16]. There are only two related studies conducted among children < 2 years old, which found that health care workers’ recommendation, parental concerns about side-effects, and parental positive attitudes toward IV were significant factors [17,18]. Hence, there is a knowledge gap about the understanding of the factors of IV for children under two years old. Such research is potentially useful for design of programs.

Furthermore, IV is free for young children in many developed countries including Canada, Slovakia, Australia, and the U.K. [19–22]. In 2008/09, the Hong Kong government recommended 6–71 months old children to take up IV annually and reimbursed HKD 80 (about USD 10) to private practitioners for IV. The market price was about HKD200 in 2011/12. The subsidy was increased to HKD 130 and HKD 160 in 2012/13 and 2014/15, respectively. Children of families receiving the Comprehensive Social Security Assistance (CSSA) scheme can take up IV free of charge at specific clinics. A study reported low IV prevalence of 9.0% among children aged 6–23 months in Hong Kong in 2006 [17]. The previous study in children IV in Hong Kong was conducted in 2006. As the government launched the subsidy policy on IV in 2008/2009, it is meaningful to conduct a study in 2011 to see if the prevalence of IV has increased over a two-year period since the implementation of the new policy. In addition, another local study, which was conducted from March to June 2011 [16], reported higher IV prevalence of 58.9% among children aged 24–59 months [17].

Behavioral health theories are commonly used to understand factors of IV. The Health Belief Model (HBM) prescribes parental perceived susceptibility and perceived severity of influenza, and perceived benefit, perceived barrier, cue to actions and self-efficacy related to IV as determinants of young children’s IV [23]. It has been used to guide variable selection in a local study among 24–59 months old children [16] and a Taiwan study investigating IV among children aged 6–36 months old [24], and other studies targeting older people, health care workers, pregnant women, and chronic diseases patients [25–29]. Given its wide applicability, the present study used it as the theoretical framework. In addition, subjective norm, defined as perceived support from significant others for performing a health-related behavior, a construct of the Theory of Planned Behaviors (TPB) [30], was relevant as family members and friends influence parental decision on children’s IV. In the local study [16], factors of IV for 24–59 months old children included family members’ IV experience, subjective norm and those related to the HBM. Other potential factors of IV included family members’ IV experience [8] and exposure to influenza-related media messages [8,31]. Fear experienced during the H1N1 pandemic was also potentially important, as fear induced from SARS/H1N1 was associated with intention to take up IV [32,33]. H1N1 has become a major source of seasonal influenza. The present study considered such factors.

We investigated prevalence and associated factors of IV among Chinese children aged 12–23 months in Hong Kong. Besides socio-demographics, potential factors included: 1) family members’ IV status, 2) parental perceptions of the HBM and subjective norm of the TPB, 3) parental fear experienced during the H1N1 pandemic, and 4) exposure to related mass media messages.

## Methods

### Study design

This cross-sectional study interviewed Chinese-speaking parents with children aged 12–23 months during July-September 2011 in Hong Kong, China. Within each of the three geographic regions (i.e. Hong Kong Island, Kowloon and the New Territories), four Maternal and Child Health Centers (MCHC) were randomly selected. The sample included parents of children aged 12–23 months who visited one of these 12 selected (out of a total of 31) MCHCs during the study period and were eligible for receiving governmental subsidy (i.e., a permanent resident of Hong Kong). During the 2010/2011 influenza season, the influenza vaccines and the vaccination program for 6–71 months old children were available in Hong Kong from November 2010 to March 2011 [12]. We included children aged 12 to 23 months, as all children of such age interval could potentially take up IV during that IV season. Furthermore, another independent study [16] was conducted among parents of children aged 24–59 months in March-June 2011, using the same questionnaire but another sampling method, allowing us to make indirect comparisons between children aged 12–23 months and 24–59 months.

The nurses of the MCHC performed eligibility screening and referred prospective participants to approach our interviewers, who briefed them about the study and invited them to join the study on-site. Participation was voluntary, and refusals would not affect participants’ right to receive any services. With written informed consent, anonymous face-to-face interviews of about 15 minutes were administered in a private room. If both parents were available, the mother was interviewed.

We estimated the sample size based on an IV prevalence rate of 9.0% from a previous study on children’s IV [17]. With a confidence level of 95% and a margin of error of 3%, the calculated sample size required is at least 350. Data obtained from 489 eligible participants were analyzed. The same study design was used in the 2006 local survey conducted for children aged 6–23 months [17]. Ethics approval was obtained from the Ethics Committee of the Department of Health and the Survey and Behavioral Research Ethics Committee of the Chinese University of Hong Kong.

### Measures

The questionnaire was constructed by a panel, considering results of a pilot study (n = 30; average Kappa for its items’ test-retest reliability [2-week apart] = 0.66).

#### Background and IV-related information

Such information included (1) background characteristics including parent participants’ and their child’s socio-demographics (e.g., sex and age), (2) index child’s IV information (including his/her IV status of any past vaccination and details of IV experience), and (3) family members’ IV experience (last 12 months).

#### Cognitive factors

HBM-related questions included two on perceived child’s susceptibility for contracting influenza (next 12 months), two on perceived severity of influenza onto the child, five on perceived benefit, six on perceived barrier (four on safety, one on inconvenience and one on cost), five on cue to action, and two on perceived self-efficacy for IV. Two questions assessed subjective norm for the child’s vaccination. The same questions were asked in a local study on IV among 24–59 month old children [16]; references were also made to other IV studies [17,34,35]. The participants rated the items on perceived susceptibility and perceived severity on 11-point scales (0 to 10), with a lower score indicating lower level of perceived susceptibility/severity regarding influenza; responses to other HBM-related items included “agree”, “disagree” and “uncertain”; those on subjective norm were rated on 4-point Likert scales (“totally disagree” to “totally agree”). Summative scales were constructed. A higher score in the items or summative scales represented a higher level of the corresponding construct.

#### Other questionnaire items

Four items assessed knowledge on governmental recommendations and IV policy. A sample item was “Does the Hong Kong government recommend children of your child’s age to take up IV?”). The question was “How fearful were you during the H1N1 pandemic?” (1 = “not at all” to 4 = “highly fearful”). Frequency of exposure to media messages about IV promotion and influenza-related severe complications/deaths among children in the past three months were also assessed on 5-point scales from “none” to “very frequently”.

### Statistical analysis

Three sets of logistic regression analyses were used to estimate the effect size and significance of the association between risk/protective factor and the binary outcome variable (i.e., IV status), with odds ratios (OR) reported (OR<1 means a negative association, OR>1 is a positive association, and OR = 1 means no association). The preliminary analysis estimated univariate odds ratios (ORu) without any statistical adjustment. The second set of logistic regression analysis for each of the independent variables (cognitions about IV) then adjusted for potential confounders (i.e., background factors in this case), and reported adjusted odds ratios (AOR). The third set of analysis fitted a multivariate backward logistic regression analysis with entry and removal criteria set at p>0.1 and p<0.05, respectively. It considered all significant variables found in the adjusted analysis as initial candidate variables and controlled for the significant background factors; the odds ratios of these models were known as multivariate odds ratios (ORm). While the adjusted analysis looked at one cognitive variable at a time, the multivariate analysis considered a number of variables simultaneously in the same model, and is hence able to suggest whether these variables have independent effects on the dependent variable, after adjusting for the other independent variables that are present in the same model. Respective 95% confidence intervals (95% CI) were derived for all odds ratios. SPSS 16.0 for Windows was used for data analyses, with statistical significance set at the 0.05 level.

## Results

### Background characteristics

About half of the index children were male; 47.4%, 51.1% and 1.4% were 12–15, 16–19, and 20–23 months old, respectively (Table 1). Majority were born in Hong Kong (99.2%), with both parents being Hong Kong residents (89.6%); 61.9 were the first child. Majority of the participants was mother of the child (80.6%) and had attained senior high or above education (90.0%); 67.7% were 30–39 years old; 55.6% were employed full-time; 3.1% of the participants’ families were covered by the CSSA. About one-fourth (26.4%) reported that their family members’ had taken up IV in the last year.

**Table 1:** Characteristics of the index children and their parent participants (N = 489).

	n	%
**Information about the index child**		
Relationship between the participant and the child		
Father	95	19.4
Mother	394	80.6
Gender of the child		
Male	265	54.2
Female	224	45.8
Age of the child (months)		
12~15	232	47.4
16~19	250	51.1
20~23	7	1.4
The child was born in HK		
Yes	485	99.2
No	4	0.8
Parity of the child		
First	302	61.8
Second	156	31.9
Third / Fourth	31	6.3
The child had ever visited a family doctor		
Yes	357	73.0
No	132	27.0
The primary care-taker of the index child		
Parent	275	56.2
Grandparent	118	24.1
Maid / Others	96	19.6
The child has ever been infected with influenza		
Yes	200	40.9
No / Refused to answer	289	59.1
The child has ever taken IV		
Yes	56	11.5
No / Refused to answer	433	88.5
*If yes*, *any side effect(s) experienced (n = 56)*		
Fever	10	17.9
Rash	1	1.8
Moderate side effects	1	1.8
*If yes*, *the IV was involved with… (n = 56)*		
Governmental subsidy–Yes	36	64.3
—No / Refused to answer	20	35.7
Private clinics—Yes	39	69.6
—No / Refused to answer	17	30.4
**Information about the parent participant**		
Relationship with the index child		
Mother	394	80.6
Father	95	19.4
HK residency status of participant and spouse		
Both	438	89.6
Either one	51	10.4
Age *		
18~29	89	18.2
30~39	331	67.7
≥40	68	13.9
Highest education level attained*		
Junior high school or below	48	9.8
Senior high school or above	440	90.0
Employment status*		
Full-time	272	55.6
Part-time / Unemployed	216	44.2
Receiving CSSA		
Yes	15	3.1
No / Refused to answer	474	96.9
Any family member has taken up IV in the last year		
Yes	129	26.4
No / refused to answer	360	73.6

*One parent participant did not answer these three questions so the total number does not add up to 489

### Child’s IV experience

Only 11.5% (95% CI = 8.5%, 14.2%) of the children had ever taken up IV. Among the 56 vaccinated children, 64.3% and 69.6% involved governmental subsidy and private clinics respectively; 10 (17.9%) were suspected to have experienced some side effects (fever: n = 10; rash: n = 1; moderate side effects: n = 1).

### Perceptions and knowledge

Of the participants, 7.5% and 45.2% perceived high susceptibility (score ≥6) for their child to contract H1N1 and seasonal influenza, respectively; 28.4% and 19.1% perceived high severity of H1N1 and seasonal influenza, respectively. Over half (57.1% to 68.1%) perceived some benefits of IV; 43.1% believed that the currently available influenza vaccines were more effective than the previously available ones. Concerning perceived barriers, prevalence of items reflecting safety concerns ranged from 35.0% (“This vaccine is not safer than the previous one”) to 58.5% (“My child is too young to take up IV”); concern for barriers of cost and inconvenience was less common (23.7% and 16.4%). Regarding cue to action, only 12.9%-19.2% had received an advice concerning the child’s IV from healthcare providers, peer parents, and relatives/friends; 17.2% believed that it was common for children of similar age to take up IV; 48.3% believed in the governmental recommendation on IV for young children. Perceived self-efficacy was very high (90.6% and 92.8%). Regarding subjective norm, less than half found their spouse (42.9%) and other family members (40.7%) supportive of the child’s IV (Table 2).

**Table 2:** Parental perceptions and knowledge toward influenza and IV.

	n	%
**(1) HBM variables**		
Perceived susceptibility and severity (Scored 6~10)		
Perceived Susceptibility Score of H1N1	37	7.5
Perceived Susceptibility Score of seasonal flu	221	45.2
Perceived Severity Score for H1N1#	139	28.4
Perceived Severity Score for seasonal flu^a^	93	19.1
Perceived benefits (Agree)		
IV is effective in preventing H1N1 influenza	279	57.1
IV is effective in reducing risk of H1N1 influenza-induced complication	314	64.2
IV is effective in preventing other seasonal influenza (not H1N1)	316	64.6
IV is effective in reducing risk of other seasonal influenza-induced complication	333	68.1
Current flu vaccine is more effective than previously available ones	211	43.1
Count for above five questions answering “agree”		
0	63	12.9
1~2	122	24.9
3~5	304	62.2
Perceived barriers (Agree)		
*(a) Safety concerns*		
This vaccine is not safer than the previous one	171	35.0
The side-effect of this vaccine is more severe	201	41.1
My baby is too young to take up this flu vaccine	286	58.5
This flu vaccine would have negative effect in interaction with other vaccines to be taken up by the baby	206	42.1
Count for the above four questions answering “agree” about perceived barriers		
0	119	24.3
1~2	202	41.3
3~4	168	34.4
*(b) Inconvenience*		
The place and time for taking up the flu vaccine is not convenient	80	16.4
*(c) Cost*		
The charge for taking up the flu vaccine is relatively high	116	23.7
Cue to action (Agree)		
Some health care providers have advised your baby to take up this flu vaccine	94	19.2
Other parents have ever given advice for your baby to take up this flu vaccine	63	12.9
Relatives or friends have ever given advice for your baby to take up this flu vaccine	64	13.1
Many parents have arranged their baby of similar age to take up this flu vaccine	84	17.2
Believe in governmental recommendation	236	48.3
Count for above five questions answering “agree”		
0	184	37.6
1~2	240	49.1
3~5	65	13.3
Perceived self-efficacy (Agree)		
You would be able to let your baby take up the vaccine if you desire to do so	443	90.6
It is your couple that decides whether your baby would take up this IV	454	92.8
Count for above two questions answering “agree”		
0	6	1.2
1	69	14.1
2	414	84.7
Subjective norms (Agree)		
Spouse supports baby taking up the vaccine	210	42.9
Other family members support baby taking up the vaccine	199	40.7
Count for above two questions answering “agree”		
0	260	53.2
1~2	229	46.8
**(2) Knowledge**		
Knowledge about governmental policies and recommendation related to IV ^c^		
Hong Kong government advises children of similar age to take up influenza vaccine	368	75.3
Vaccine currently provided by the government for children (aged 6–72 months) includes H1N1 vaccine	117	23.9
Hong Kong government recommends current vaccine that includes H1N1 to children of similar age	196	40.1
WHO recommends current vaccine that includes H1N1 to children of similar age	137	28.0
Count for number of above five questions answering “yes”		
0	88	18.0
1~2	257	52.6
3~4	144	29.4
Knowledge about influenza vaccine requirement ^*c*^		
Children (aged 6 months to less than 6 years) should take up influenza vaccine every year	290	59.3
**(3) Other factors**		
Emotional responses to H1N1		
Moderate / high fear during the H1N1 pandemic ^b^	234	47.9
Exposure to media information in the last 3 months ^*c*^		
Obtained information about severe complications and deaths of influenza from	134	27.4
Exposed to promotion on IV from the media	268	54.8

# 8 participants answered “do not know”

^a^ 3 participants answered “do not know”

^b^ 1 participant answered “do not know”

^c^ Only percentages of participants answering “yes” or “often / frequently” in the above item were presented in this table

Majority of the participants knew that the local government advised children of similar age to take up IV (75.3%); 40.1% and 28.0% knew that the local government and WHO recommended current influenza vaccines including H1N1 to children of similar ages; about one-fourth knew that the vaccines currently provided by the local government for children included the H1N1 vaccine (23.9%). Only about two-third knew that children aged 6–71 months should take up IV annually (59.3%) (Table 2).

### Fear and media exposure

About half (47.9%) had experienced moderate to severe levels of fear during the H1N1 pandemic. In the last three months, 27.4% had often/frequently been exposed to media messages depicting severe complications and/or deaths caused by influenza among children; 54.8% had often/frequently been exposed to media messages promoting IV (Table 2).

### Factors of IV

Child’s age was the only significant background factor (16–19 months: ORu = 2.88, 95%CI = 1.52, 5.46; 20–23 months: ORu = 11.68, 95%CI = 2.38, 57.36; Table 3); it was adjusted for in subsequent analyses. A number of variables were significantly associated with IV status and in both the univariate and adjusted analyses (Table 3). Their AOR were: (1) family members’ IV experience in the last 12 months (AOR = 6.60, 95% CI = 3.60, 12.11), (2) knowledge about governmental policy and recommendations on IV for young children (3–4 correct responses: AOR = 2.64, 95% CI = 1.09, 6.40; reference: no correct response), (3) knowledge about the annual vaccination requirement (AOR = 2.30, 95% CI = 1.21, 4.38), (4) the HBM-related variables [perceived barrier related to safety (1–2 item responses: AOR = 0.24, 95% CI = 0.12, 0.47; 3–4 item responses: AOR = 0.14, 95% CI = 0.06, 0.33; reference: 0 item response), and cue to action (3–5 item responses: AOR = 7.79, 95%CI = 3.45,17.58; reference: 0 item response)], and (5) supportive subjective norm (AOR = 4.59, 95% CI = 2.34, 9.00). Two other variables, influenza experience (ORu = 1.79, 95% CI = 1.02, 3.12) and perceived severity of seasonal influenza (ORu = 0.38, 95% CI = 0.15, 0.98) were significant in the univariate analysis but not in the adjusted analysis.

**Table 3:** Factors associated with IV (ever received) of the index child (among all participants).

	IV (Ever Received)			
	n	Row (%)	ORu (95%CI)	AOR (95%CI)
All	489	11.5		
Age of the child (months)				
12~15	232	6.0	1.0	
16~19	250	15.6	2.88(1.52,5.46)**	
20~23	7	42.9	11.68(2.38,57.36)**	
Index child ever been infected with influenza				
No / Do not know	289	9.0	1.0	1.0
Yes	200	15.0	1.79(1.02,3.12)*	1.59(0.90,2.83)
Perceived severity of seasonal influenza#				
Low (scored 0–5)	393	13.0	1.0	1.0
High (scored 6–10)	93	5.4	0.38(0.15,0.98)*	0.40(0.15,1.05)
Family members ever taken up IV in the past year				
No / Do not know	360	5.6	1.0	1.0
Yes	129	27.9	6.58(3.64,11.90)**	6.60(3.60,12.11)**
No. of correct responses reflecting knowledge about governmental policy and recommendation				
0	88	8.0	1.0	1.0
1~2	257	7.8	0.98(0.40,2.39)	0.86(0.34,2.14)
3~4	144	20.1	2.92(1.22,6.99)*	2.64(1.09,6.40)*
Knowledge about annual vaccine requirement				
No / Not sure	199	7.0	1.0	1.0
Yes	290	14.5	2.24(1.19,4.22)*	2.30(1.21,4.38)*
No. of item responses reflecting perceived safety barriers				
0	119	27.7	1.0	1.0
1~2	202	7.4	0.21(0.11,0.41)**	0.24(0.12,0.47)**
3~4	168	4.8	0.13(0.06,0.30)**	0.14(0.06,0.33)**
No. of item responses reflecting cues to action				
0	184	6.0	1.0	1.0
1~2	240	9.6	1.67(0.79,3.51)	1.63(0.76,3.46)
3~5	65	33.8	8.05(3.63,17.86)**	7.79(3.45,17.58)**
No. of item responses reflecting supportive subjective norms				
0	260	4.6	1.0	1.0
1~2	229	19.2	4.92(2.53,9.57)**	4.59(2.34,9.00)**

*p<0.05

**p<0.01. ORu: univariate variables significantly associated with ever received influenza vaccination

AOR: odds ratio adjusted for age of the child because only one significant background variable (i.e., child’s age) was shown here and controlled in the adjusted analyses

Cognitive variables that were not significant in univariate analyses included: perceived susceptibility of H1N1 and seasonal influenza, perceived severity of H1N1, perceived benefits, inconvenience and cost items of taking up IV, perceived self-efficacy, fear during H1N1 pandemic and the two items about media exposure; # 3 missing cases.

Using all the significant variables found in the adjusted analysis as initial variables, three independent variables were kept by the stepwise backward multivariate logistic regression model, while the rest were removed from the final multivariate model. The variables of the model included family members’ IV experience (ORm = 6.24, 95% CI = 3.20, 12.18), the scale on perceived barrier related to safety of IV (1–2 item responses: ORm = 0.29, 95% CI = 0.13, 0.62; 3–4 item responses: ORm = 0.23, 95% CI = 0.09, 0.60; reference group: 0 item response), and supportive subjective norm (ORm = 2.37, 95% CI = 1.02,5 .53). Such results are not shown in the tables.

## Discussion

Based on the data of 489 Chinese parents of children aged 12–23 months, the results showed that only 11.5% of the children had ever received IV. The significant factors of the children’s IV included knowledge about the governmental policy/recommendation, knowledge about annual IV requirement, perceived safety-related barrier, cue to action and subjective norm.

More countries/regions are providing full financial support than providing partial financial support (e.g., Latvia and Hong Kong) for IV among young children [19,36]. Despite introduction of the local subsidization policy in 2008/09, we found that IV prevalence among very young children (aged 12–23 months) remained very low in 2011 (11.5%). As a comparison, IV prevalence was 9.0% among 6–23 months old children (and 9.3% among 12–23 months old children) in the 2006 study [17] that used same sampling and data collection methods as those of this study. Thus, improvement in coverage of IV in the 12–23 months group over the five years was minimal. The prevalence was much lower than that of children aged 6–23 months in the U.S. (56%) and France (21%) [13,37]. Thus, the subsidization policy in Hong Kong and related health promotion did not seem to have increased coverage of IV in this age group substantially. Cost was not a barrier in this age group, as only 23.7% perceived that cost for IV was relatively high and perceived cost was not a significant factor of IV. The non-significance of cost suggests that the sampled parents were not responsive to the subsidization policy.

An important insight comes from the very strong positive association between the child’s age and IV uptake observed in this study. It seems that many parents did not object to IV for their children, but instead, postponed their action, as 58.9% of the children aged 24–59 months had taken up IV in 2011(during approximately the same study period as this one) [16] versus 11.5% of the 12–23 months old children involved in this study. Also, 58.5% of the parents perceived that their children were too young to take up IV. The concern of the child’s age might be due to worry about side-effects of IV. In both this study and the 2006 survey targeting children aged <2 years [17], perceived safety of IV was strongly associated with the child’s IV status; the findings also corroborate those of other studies [16,17,24]. Young children’s parents need to know that side-effect of IV among young children is not age dependent and that children aged 12–23 months need as much protection as older children [3], and as early as possible.

This study, the 2006 survey (6–23 months old children) [17], and other studies targeting young children [16,24] consistently found strong associations between health care workers’ advice and the child’s IV status. The adjusted analysis of both this and the independent concurrently conducted survey in a 24–59 months group [16] found very strong associations between cue to action (including that given by health professionals) and IV status. About 20% of the participants had been advised about their children’s IV, which was almost doubled when compared to the data (10.6%) obtained from a comparable survey conducted in 2006 [17]. The prevalence of this study was still low, despite improvement. It is important as about 90% of the local newborns would visit MCHC [38]. Thus, health care workers need to play a stronger role in information dissemination and promotion of IV for children under two years old. Furthermore, about 24.7% did not know that IV has been recommended for children aged 6–71 months, while that knowledge was a significant factor of IV. The government should also promote the knowledge that IV is required annually for young children, which was another significant factor of IV in this study.

In this study, about 40% of the indexed children had been infected by influenza but the experience was not associated with IV status. It implies that prior experience of influenza cannot motivate parents to vaccinate their children. On the other hand, both family members’ IV experience and supportive subjective norm (of spouse and family members) were significant in both the adjusted and stepwise analyses. The independent concurrently conducted study [16] also found significant associations between these two factors and IV among children aged 24–59 months. Although IV among young children is a family matter, only half of the spouse and family members were supportive of the child’s IV both in this and the 24–59 months group studies [16]. As a new approach, counseling is needed to harmonize divergent opinions about children’s IV and to enhance communication skills among parents. The significant composite variable on cue to action found in this and the 24–59 months group studies [16] included items that involved friends, relatives, and other peer parents. We should hence engage parents’ social networks, possibly through social media or other means. Pilot studies are warranted to take this new approach.

Only two HBM constructs (perceived barriers and cue to action) were found to be significant factors; perceived susceptibility and perceived benefits were significant in the concurrently conducted local study of the 24–59 months group [16] but not in this study’s 12–23 months group. Perceived severity and perceived efficacy were non-significant in both studies. Likewise, perceived severity and perceived benefit were not significantly associated with the child’s IV experience in the 2006 study (6–23 months group) [17]. Although HBM is applicable to understand IV in different high risk groups [26,27], it seems to be working less well in explaining IV among children aged 12–23 months. Making IV more convenient and less costly might not be effective in promoting IV, as perceived efficacy and cost as perceived barriers were not significant factors in this study’s age group. Related media exposure was non-significant in both this study and the concurrently conducted 24–59 months group local study [16], suggesting general media campaigns are not promising. Fear experienced during the H1N1 pandemic was a significant factor for the 24–59 months group [16] but not for this study’s age group. Explanation of the difference is beyond the scope of this report. The observation that most of the local health promotion campaigns for IV among young children have been engaging some of the aforementioned non-significant components might partially explain the low IV prevalence in this age group.

The study has the strength of having some degree of comparability with two other local studies involving young children below [17] and above two years old [16]. It also has some limitations. It did not include children who were 6–11 months old during the study period (July-September 2011), as those children were younger than six months during the 2010/2011 IV season (November 2010 to March 2011) and were not by then recommended to take up IV. We contended that IV prevalence of the 6-11-month age group was even lower than that of the 12–23 month group, as IV was positively associated with age. Given that 92% of the Hong Kong population are Chinese [39], we only investigated the IV among Chinese children. Findings of other ethnic groups might be different and require another study. Also, the study’s response rate was not measured. The nurses were engaged in clinical work and did not have time to record such data. Furthermore, the 10% of the newborns who did not visit MCHC were not included in this study. We did not interview both parents but just one parent of the index child because usually only one parent visited the MCHC with the child. The scales were constructed by the research team, although they were almost identical to those used in a published study for the 24–59 months group [16]. Data were self-reported and might involve reporting bias. Our data were collected in 2011; future studies are recommended to use this study’s data to construct trends. Moreover, the current findings cannot infer causality, as they are cross-sectional in nature.

To conclude, prevalence of IV among 12–23 months old Chinese children in Hong Kong was low and showed little improvement as compared to 2006. Parents might be postponing these children’s IV unnecessarily. Effectiveness of the governmental recommendation, subsidy, use of some HBM constructs and mass media in increasing such children’s IV is uncertain. The government should revamp related health promotion strategies and treat the young age group (12–23 months) as a distinct segment. It should also emphasize dissemination of governmental policies, increase perceived safety of IV for these children, and solicit support from primary care doctors, family and friends. Children aged 12–23 months in Hong Kong were not well protected against influenza and comprehensive strategies are needed.
